# Antiaging Active Natural Products

**DOI:** 10.1155/2013/685675

**Published:** 2013-03-07

**Authors:** Yukihiro Shoyama, Chun-Su Yuan, Daofeng Chen, Tibor Wenger

**Affiliations:** ^1^Department of Pharmacognosy, Faculty of Pharmaceutical Science, Nagasaki International University, Sasebo, Nagasaki 859-3298, Japan; ^2^Pritzker School of Medicine, University of Chicago Medicine, 5841 S. Maryland Avenue, Chicago, IL 60637, USA; ^3^School of Pharmacy, Fudan University, 138 Yixueyuan Road, Shanghai 200032, China; ^4^Department of Human Morphology, Semmelweis University, H-1085 Budapest, Hungary

Many diseases related to aging have been increasing because the aged population rapidly increases in the world. Therefore, the importance of antiaging active natural products has been increasing. Aging brings many disorders like cardiovascular problems, diabetes, senile dementia, Alzheimer's disease, cerebral infarction, degenerative neural disease, sleeping problems, various kinds of cancer, and so on. Since the side effects of modern medicines for aged bodies frequently occurs, the therapeutic use of natural products like herb medicines and/or phytomedicines having antiaging activity has been expanding widely depending on the accumulation of evidence-based experiences. As traditional medicines have a historical background of thousands of years based on a huge number of experiments for human bodies, such real evidence-based traditional medicine becomes important to accumulate the knowledge of antiaging activities.

 The authors in this special issue provide a comprehensive summary of their most recent knowledge on antiaging natural products. The more than 60 plant species including several pure compounds have been introduced in this issue. Also 500 or more references found at the end of individual chapters will help not only antiaging researchers but also many researchers, research scholars, academician, industrialists, and so on, for working in the numerous fields of natural products. This issue has encompassed all branches of antiaging phenomena like skin aging, hepatoprotective, antioxidant activity, Alzheimer's disease, cerebral ischemia, memory impairment, and brain damage, neuroprotection, inflammation, and anticancer. Therefore, this issue is widely related to pharmacognosy, pharmacology, pharmaceutical chemistry, natural product research, clinical, and hospital pharmacy and cosmetics and so on. All interested readers around the world can freely access articles online.

*Yukihiro Shoyama* *Yukihiro Shoyama* *Chun-Su Yuan* *Chun-Su Yuan* *Daofeng Chen* *Daofeng Chen* *Tibor Wenger* *Tibor Wenger*

